# Exploring the Evidence for Personalized Pharmacotherapy in Type 2 Diabetes—A Systematic Review

**DOI:** 10.3390/jpm15110539

**Published:** 2025-11-06

**Authors:** Velimir Altabas, Jelena Marinković Radošević

**Affiliations:** 1 Department of Endocrinology, Diabetes and Metabolic Diseases, Sestre Milosrdnice UHC, 10000 Zagreb, Croatia; 2 School of Medicine, University of Zagreb, 10000 Zagreb, Croatia

**Keywords:** metformin, thiazolidindiones, DPP-4 inhibitors, GLP-1RAs, insulin, SGLT-2 inhibitors, miRNA

## Abstract

**Background/Objectives**: Type 2 diabetes mellitus (T2DM) is a complex metabolic disorder characterized by insulin resistance, impaired insulin secretion, and chronic hyperglycemia. Recent studies have identified microRNAs (miRNAs), a class of small non-coding RNAs that regulate gene expression at the post-transcriptional level, as modulators of pathways involved in T2DM pathophysiology. Dysregulated miRNA expression has been detected in various samples collected from patients with T2DM, implicating these molecules in disease onset and progression. **Methods**: We systematically searched PubMed, Scopus, and Web of Science for studies published from the earliest available records to 18 August 2025 using the following Boolean search terms: “miRNA AND gliclazide”, “miRNA AND glibenclamide”, “miRNA AND gliquidone”, “miRNA AND glimepiride”, “mirRNA AND metformin”, “miRNA AND pioglitazone”, “miRNA AND rosiglitazone”, “miRNA AND sitagliptin”, “miRNA AND vildagliptin”, “miRNA AND alogliptin”, “miRNA and saxagliptin”, “miRNA AND linagliptin”, “miRNA AND liraglutide”, “miRNA and dulaglutide”, “miRNA AND semaglutide”, “miRNA AND tirzepatide”, “miRNA AND lixisenatide”, “miRNA AND empagliflozin”, “miRNA AND dapagliflozin”, miRNA AND insulin glargine”, “miRNA AND insulin detemir”, “miRNA AND insulin degludec”, “miRNA AND insulin aspart”, “miRNA AND insulin glulisine”, and “miRNA AND insulin lispro”. Additionally, gray literature was searched in ClinicalTrials.gov, the EU Clinical Trials Register (EudraCT), and the ISRCTN Registry to identify unpublished studies. Studies were eligible for inclusion if they were clinical interventional studies assessing the impact of currently available antidiabetic treatments on miRNA expression. Only articles published in English were considered. The risk of bias was evaluated using the RoB2 (Risk of Bias 2) and ROBINS-I (Risk Of Bias In Non-randomized Studies—of Interventions) tools. Study characteristics and major findings were tabulated. **Results**: A total of 1263 manuscripts was identified initially. After removing duplicates, 726 articles remained for further screening. Ultimately, 17 manuscripts reporting interventional clinical trials on the effects of antidiabetic treatment on miRNA were included, encompassing a total of 1093 patients. Key findings included treatment-associated changes in miRNA expression and their potential utility for the prediction of clinical outcomes. **Conclusions**: Current evidence supports the hypothesis that antidiabetic treatments modulate miRNA expression, with some findings showing predictive value for metabolic outcomes. However, the available data remain limited and of low grade of certainty, and further large-scale clinical studies are needed to provide deeper insights into these associations.

## 1. Introduction

Diabetes mellitus comprises a group of distinct chronic metabolic diseases with diverse—and in many cases not fully understood—etiologies, specific pathophysiological mechanisms, and diverse treatment approaches. Based on the differences in etiology and clinical characteristics, diabetes is currently classified into type 1 diabetes, type 2 diabetes, and gestational diabetes mellitus, along with other specific forms caused by genetic mutations, endocrine disorders, exocrine pancreatic disease, or medication use [1,2].

Diabetic complications may arise acutely or develop gradually, contributing to both short-term and long-term morbidity and mortality. Globally, in both developed and developing countries, chronic diabetic complications are the leading cause of blindness, renal failure, and non-traumatic lower limb amputations. Diabetes is acknowledged as a major risk factor for cardiovascular disease and stroke [3,4]. Maternal diabetes during pregnancy can result in adverse outcomes for the child [5,6]. In addition, diabetes is increasingly recognized as a risk factor for cognitive decline, functional disability, affective disorders, obstructive sleep apnea and liver disease [7].

The defining clinical characteristic of all types of diabetes is disturbed carbohydrate metabolism, leading to chronic hyperglycemia, whose severity is directly associated with the prevalence and progression of diabetic complications [1,8,9]. The mechanisms underlying hyperglycemia vary among the different types of diabetes and may involve impaired insulin secretion from the pancreas, reduced glucose utilization in tissues, increased hepatic gluconeogenesis and glycogenolysis, hormonal alterations during pregnancy, and other contributing factors [10,11,12]. Regardless of the specific underlying metabolic disturbance, the clinical diagnosis of diabetes is based on the laboratory evidence of elevated blood glucose levels and increased glycated hemoglobin (HbA1c) [1].

Today, type 2 diabetes mellitus (T2DM) is the most prevalent form of the disease, accounting for nearly 90% of patients worldwide [13]. Of further concern, according to the International Diabetes Federation, nearly 11% of the global adult population is diagnosed with diabetes [14]. The figure is still rising, particularly for type 2 diabetes [15].

Furthermore, type 2 diabetes mellitus is increasingly recognized as a heterogeneous group of disorders driven by diverse additional pathophysiological mechanisms. These additional contributing factors include age, obesity, and disordered eating, alterations in gut microbiota, impaired incretin secretion, and aberrant expression of sodium–glucose cotransporters, among others. Based on differences in the etiology of type 2 diabetes, some authors have proposed further subclassification of type 2 diabetes phenotypes at the time of diagnosis, including, but not limited to, mild obesity related phenotypes, mild age-related phenotypes, severe insulin-insufficient phenotypes, and severe insulin-resistant phenotypes [16]. In clinical practice, these phenotypes are further complicated by the presence of diabetic complications, which can substantially influence treatment strategies [17].

However, effective treatments are available, including patient education, empowerment, nutritional therapy, physical activity, and pharmacological interventions. The ultimate goals are normalization of blood glucose and the reduction in the risk of chronic diabetic complications, including cardiovascular and cerebrovascular disease. Contemporary therapies rely on a “treatment-to-target” approach, making diabetes management a highly individualized process. Given the wide spectrum of available pharmacotherapeutic agents, specific national and international guidelines provide recommendations for a structured treatment approach tailored to different patient phenotypes [18,19].

Nevertheless, long-term treatment outcomes remain unpredictable for individual patients and depend on factors such as genetics and epigenetic modifications, post-translational and other specific pathophysiologic mechanisms involved, and treatment availability and adherence [12,20,21,22,23]. Therefore, it is not surprising that many patients with type 2 diabetes mellitus receive suboptimal treatment, thereby increasing their risks of progression of diabetic complications, disability, and reduced life expectancy. The overall societal burden and costs of suboptimal diabetes treatment should not be overlooked. Current antidiabetic treatments are often resource-intensive and expensive, raising concerns about cost-effectiveness, equitable access, and the potential to exacerbate healthcare disparities [24,25,26]. Consequently, there is a pressing need to advance predictive and precision medicine approaches to optimize current treatment strategies, improve patient outcomes, and allocate healthcare resources more efficiently [27].

Recent studies on genetics and epigenetics in type 2 diabetes mellitus have provided valuable insights into interindividual variability in disease onset, progression, and treatment response [28,29,30]. However, it remains under debate whether this research can be fully translated into clinical practice in humans. Human genetic material has remained relatively stable throughout evolution, and changes may lead to evolutionary shifts. Consequently, the concept of gene therapy for type 2 diabetes raises not only technical but also ethical challenges [31]. Similarly, epigenetic modifications such as DNA methylation, histone modification and chromatin remodeling alter gene expression without changing the original DXA sequence. These modifications have been linked to impaired insulin secretion and increased insulin resistance, impaired glucose metabolism, and inflammation. Importantly, even relatively modest environmental factors like diet, overweight, and physical inactivity can induce epigenetic changes, explaining the link between lifestyle and disease progression [32,33]. However, certain epigenetic changes can also be transmitted to offspring, potentially influencing the disease risk across generations, and thus limiting the therapeutic potential of this approach. In addition, concerns about possible discrimination, patient re-identification, and unexpected findings may arise [34].

By contrast, microRNAs (miRNAs) are small non-coding RNAs that regulate gene expression post-transcriptionally, representing another layer of gene regulation. They target the messenger RNA and may induce its degradation or function loss, thereby modulating protein synthesis and gene expression [35]. Dysregulated miRNAs have been linked to impaired insulin secretion, insulin resistance, and chronic inflammation in type 2 diabetes [36,37]. Several miRNAs, including miR-375, miR-29, miR-34a, and miR-103/107, have been involved in pancreatic β-cell apoptosis, impaired insulin signaling, lipid metabolism abnormalities, and cardiovascular disease [38,39,40,41,42].

The therapeutic impact of miRNAs in type 2 diabetes mellitus could encompass several distinct approaches. Some agents could act as miRNA antagonists (also called antagomirs, or miRNA inhibitors), which inhibit the activity of overexpressed deleterious miRNAs. Others may act as miRNA agonists or mimics (agomirs), restoring the function of beneficial miRNAs and thereby reestablishing physiological regulation of glucose metabolism [43]. In addition, miRNA activity can be modulated by already existing antidiabetic therapies, suggesting that at least a part of the therapeutic effect of currently prescribed antidiabetic drugs may be mediated through alterations in miRNA expression [44].

These directions could imply a novel approach to diabetes care, as novel biomarkers for early diagnosis, risk stratification, and prediction of treatment response are actively investigated, and they may contribute to the development of personalized therapeutic strategies in type 2 diabetes. However, current treatments should not be overlooked, as a growing body of evidence suggests that at least some of these agents possess miRNA modulatory properties, which may partly mediate their clinical effects.

This systematic review aims to evaluate the current evidence regarding the impact of contemporary drug treatments for type 2 diabetes mellitus on miRNA expression, with a particular focus on the potential, yet not fully understood, role of miRNA in modern diabetes pharmacotherapy.

## 2. Materials and Methods

In preparation for this systematic review, the guidelines specified in the PRISMA2020 statement: An updated guideline for reporting systematic reviews were adhered to [45]. This review is registered in the PROSPERO database (PROSPERO registration number: 2025 CRD420251140020).

### 2.1. Eligibility Criteria

Studies eligible for inclusion in this review had to be interventional clinical studies on patients with type 2 diabetes that reported the effects of specific currently approved antidiabetic drugs on miRNAs. Only articles published in English were considered.

Manuscripts were excluded if they did not report original research on pharmacological interventional studies on patients with type 2 diabetes. Studies that focused on other types of diabetes were also excluded. Furthermore, conference papers and proceedings, preclinical studies, reviews, editorials, commentaries, letters, notes, errata, retractions, and case reports were not included.

### 2.2. Search Strategies

We conducted a comprehensive search for interventional clinical studies investigating the effects of antidiabetic drugs on miRNAs.

The search used the following Boolean terms: “miRNA AND gliclazide”, “miRNA AND glibenclamide”, “miRNA AND gliquidone”, “miRNA AND glimepiride”, “miRNA AND metformin”, “miRNA AND pioglitazone”, “miRNA AND rosiglitazone”, “miRNA AND sitagliptin”, “miRNA AND vildagliptin”, “miRNA AND alogliptin”, “miRNA and saxagliptin”, “miRNA AND linagliptin”, “miRNA AND liraglutide”, “miRNA and dulaglutide”, “miRNA AND semaglutide”, “miRNA AND tirzepatide”, “miRNA AND lixisenatide”, “miRNA AND empagliflozin”, “miRNA AND dapagliflozin”, miRNA AND insulin glargine”, “miRNA AND insulin detemir”, “miRNA AND insulin degludec”, “miRNA AND insulin aspart”, “miRNA AND insulin glulisine”, and “miRNA AND insulin lispro”. The search was performed in PubMed, Scopus, and Web of Science. Additionally, gray literature was searched in ClinicalTrials.gov, the EU Clinical Trials Register (EudraCT), and the ISRCTN Registry to identify unpublished studies. Only trials with posted or publicly available results were considered for inclusion.

Manuscripts published from the earliest available records through 18 August 2025 were included, with no filters or limits applied.

### 2.3. Study Selection and Data Extraction

The eligibility of articles was independently evaluated by two reviewers according to predefined inclusion criteria, specifically the reporting of effects of non-insulin antidiabetic drugs on miRNA. Along with outcome relevance, data on patient characteristics and follow-up length were extracted. Any discrepancies between reviewers were resolved through discussion.

Finally, all titles and abstracts meeting the inclusion criteria were retrieved for full-text review. No automation tools were employed in the selection process.

### 2.4. Risk of Bias Assessment

The risk of bias was independently determined by two reviewers using the Risk Of Bias 2 (RoB2) tool for randomized clinical trials (RTCs) and the Risk Of Bias In Non-randomized Studies—of Interventions (ROBINS-I) tool for non-randomized studies. A detailed description of these tools is available elsewhere [46,47].

### 2.5. Data Synthesis

To assess eligibility for data synthesis, the intervention characteristics of each study were first tabulated, including specific details as drug type administered, population characteristics and follow-up duration.

In cases when summary statistics were missing or incomplete, relevant data were systematically extracted from tables, figures, or supplementary materials to ensure comprehensive inclusion in the analysis.

Results from individual studies and syntheses were organized into summary tables to facilitate cross-study comparison. Key variables, including intervention type, outcome measures, and primary findings, were systematically tabulated. Because accurate measurement of miRNAs requires rigorous analytical and reporting standards to ensure reproducibility and clinical relevance, adherence to current guidelines for publication of quantitative real-time PCR experiments was also evaluated and summarized [48]. The results were calculated as the sum of the scores assigned to each MIQE 2.0 category (0 = non-compliant, 0.5 = partially compliant, 1 = fully compliant), divided by the maximum possible score and multiplied by 100%. Finally, the strength of evidence was assessed using the GRADE system [49].

All steps in data synthesis were performed independently by two reviewers, with any discrepancies resolved through discussion.

## 3. Results

### 3.1. Study Selection

**Figure 1:**
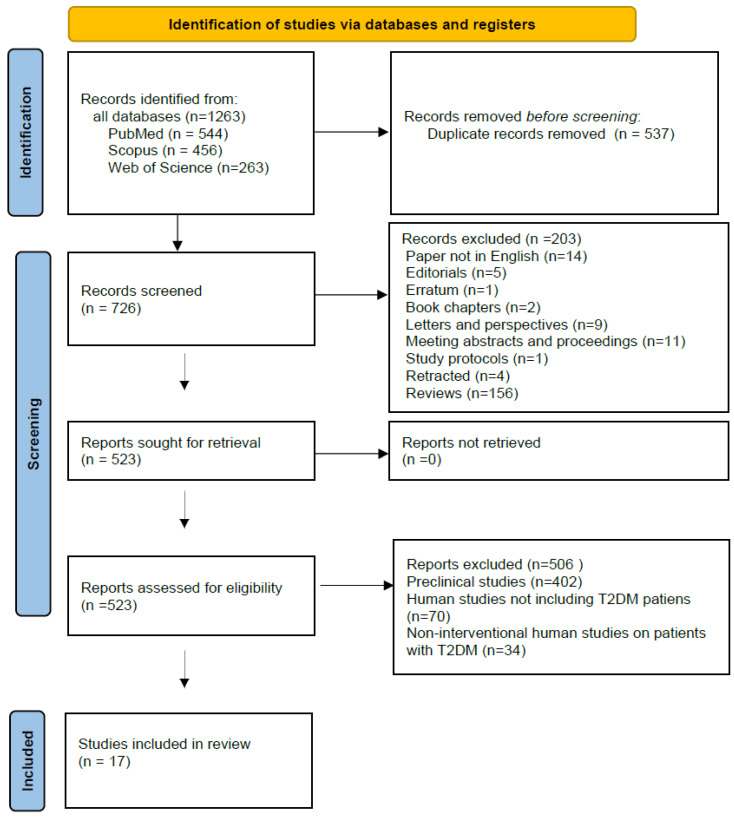
Flow chart of the sequence of steps in the collection and selection of qualified studies (PROSPERO registration number: 2025 CRD420251140020).

A total of 1263 articles were identified during the search through prespecified databases (PubMed, *n* = 544; Scopus, *n* = 456; Web of Science, *n* = 263). After removing duplicate manuscripts, 726 articles were screened. Articles written in languages other than English were excluded, as well as editorials, book chapters, errata and retracted articles, letters, perspectives, meeting abstracts and proceedings, study protocols, and reviews. After these exclusions, 523 studies on miRNA and various antidiabetic agents remained. Next, preclinical studies (*n* = 402), clinical studies not involving patients with type 2 diabetes (*n* = 70), and non-interventional clinical studies on patients with type 2 diabetes *(n* = 34) were excluded. No additional eligible studies were found in the gray literature search. Ultimately, 17 interventional studies on patients with type 2 diabetes investigating the effect of various antidiabetic drugs on miRNA were identified for full-text review. All these manuscripts were retrieved for detailed assessment. Figure 1 presents the flow diagram describing the study selection process.

### 3.2. Study Characteristics

The main finding from the search results is the limited number of relevant evidence-based studies. This may be explained by the relatively recent discoveries and technological advances, considering miRNAs that have only recently enabled such research. In contrast to the abundance of preclinical studies, clinical research in this field remains scarce.

We identified 17 interventional clinical studies investigating the effects of various antidiabetic drugs on miRNA expression in patients with T2DM (Cohen’s κ ≈ 0.91 for inter-rater agreement). Among these, three analyzed the impact of metformin, three evaluated dipeptidyl dipeptidase 4 (DPP-4) inhibitors, three examined thiazolidinediones, six assessed glucagon-like peptide 1 receptor agonists (GLP-1 RAs), three focused on sodium glucose cotransporter 2 (SGLT-2) inhibitors, and one study investigated intensified insulin treatment. In addition, two studies used sulfonylureas as comparators. Notably, some studies included multiple treatment arms investigating different antidiabetic drugs.

The study populations in these studies were diverse. While most of these studies included patients with T2DM without specified comorbidities or complications, some research focused on T2DM patients with arterial hypertension (*n* = 1), heart failure with preserved ejection fraction (HFpEF) (*n* = 1), coronary artery disease (CAD) (*n* = 2), non-alcoholic fatty liver disease (NAFLD) (*n* = 1), and diabetic kidney disease (*n* = 1).

Across the 17 included studies, all analyses used real-time quantitative polymerase chain reaction (RT-qPCR)-based platforms, with two studies also employing NanoString for broader profiling. The predominant biological sources were serum or plasma (*n* = 13), with fewer studies analyzing adipose tissue (*n* = 2), whole blood (*n* = 1), urine (*n* = 1) or extracellular vesicle fractions (*n* = 1).

Normalization strategies varied considerably among the included studies. Four studies did not report any normalization method. Two studies applied global mean normalization across profiled targets, while spike-in controls with quantile normalization were used in one study. Multiple endogenous reference miRNAs were employed in one study, and single endogenous controls—including miR-191-5p and U6 snRNA—were reported in two studies. NanoString internal normalization was applied in one study, and two studies used exogenous spike-in controls (cel-miR-39). Furthermore, for most studies, hemolysis control data were not provided. Only 1 study explicitly reported hemolysis assessment via visual inspection and evaluation of hemolysis-sensitive miRNAs, while another study applied a ratio-based molecular control. However, hemolysis assessment was not necessary for urine or tissue samples that did not contain blood.

Adherence to MIQE and MIQE 2.0 reporting standards was heterogeneous. The median MIQE 2.0 adherence across the 17 studies was 70% with a range from 35% to 90%. Sixteen of seventeen reports included information on basic methods but lacked substantial details on assay validation, pre-amplification, and reproducibility. Only three studies documented amplification efficiency and dynamic range; two reported intra-assay and inter-assay coefficients of variation. None provided data availability statements or raw Cq values, which are now recommended under MIQE 2.0 to facilitate reproducibility and transparency.

Collectively, these findings indicate that while fundamental analytical procedures are typically described, reporting of validation, normalization justification, and reproducibility metrics remains suboptimal, underscoring the need for stricter adherence to updated MIQE 2.0 guidelines in circulating and tissue miRNA studies.

Last, but not least, a variety of miRNAs were analyzed across studies, with diverse outcome measures reported.

Key characteristics and findings of the included studies are provided in Table 1.

### 3.3. Risk of Bias in Studies

A critical appraisal of the quality of the selected articles was conducted using the Risk Of Bias 2 (RoB2) tool for randomized trials and the ROBINS-I tool for non-randomized studies.

Among the 17 included studies, six randomized controlled trials were evaluated using RoB2 and demonstrated mainly low-to-moderate risk of bias, primarily due to incomplete blinding or relatively short study duration. Of the remaining 11 observational studies, assessed with ROBINS-I, seven exhibited a predominantly moderate risk of bias. The most frequent concerns involved small sample sizes, confounding, selection bias, and the lack of a control group. Serious risk of bias was identified in four studies, particularly those with small sample sizes, no control group, selective outcome reporting, or inadequate adjustment for clinical covariates.

Overall, the body of evidence is at moderate-to serious risk of bias, suggesting findings should be interpreted with caution. The generalizability of the results may also be limited due to potential genetic differences among populations from different countries. Furthermore, the clinical applicability of these findings remains uncertain. Additionally, these studies focused primarily on patients with type 2 diabetes without chronic complications, while other patient populations, such as those with cardiovascular or renal complications, were underrepresented.

The quality assessment outcomes of each included research are presented in Table 2.

Since no additional studies were identified in the gray literature search, publication bias could not be formally assessed due to the absence of unpublished trials.

### 3.4. Results of Individual Studies

According to the analyzed studies, currently available standard antidiabetic treatments do affect circulating miRNAs, and the impact varies by drug class. This suggests epigenetic modulations of glucose metabolism and potential other, yet unidentified, mechanisms of action. However, there have been differences between studies in terms of study design, population characteristics, and follow-up periods. In some studies, surrogate clinical outcomes were analyzed [50,53,54,58,59,61,65].

With respect to specific drugs and drug classes, metformin has been associated with a broad downregulation of multiple miRNAs. In contrast, studies on thiazolidinediones, DDP-4 inhibitors, GLP-1Ras, SGLT-2 inhibitors, and insulin have yielded mixed results regarding miRNA upregulation or downregulation [50,51,53,54,55,56,58,59,60,63,64,65,66]. Furthermore, research on the effects of thiazolidinediones, SGLT-2 inhibitors, GLP-1 Ras, and insulin treatment on miRNAs has also investigated associated clinical benefits [50,53,58,59,64,65]. In some studies, the predictive value of baseline levels of specific miRNAs for various clinical outcomes was also investigated [50,52,53,58,59,61,62,64,65].

Patient characteristics like age and comorbidities (CAD, NAFLD, HFpEF) may also influence which miRNAs are responsive; for instance, youth-onset vs. adult-onset T2DM is associated with different predictive markers [62].

The main findings of each research are presented in Table 1.

### 3.5. Strength of Evidence

The strength of the evidence was formally evaluated using the GRADE system and summarized for each drug and drug class. The details are provided in Table 3.

For the various drugs, the strength of evidence ranged from low to low-to-moderate. The main limitations of the studies included in this review were the relatively small number of randomized controlled trials (RCTs) with short study durations, a relatively high risk of bias, inconsistent endpoints, and limited clinical correlation.

## 4. Discussion

The interventional clinical studies included in this review suggest an emerging role of miRNAs as potential predictors of T2DM progression and treatment response. They also provide new insights into the mode of action of currently available treatments. Across diverse drug classes, different miRNAs have been associated with the age of T2DM onset, disease severity, and responsiveness to specific drug classes, highlighting their potential utility in precision and predictive medicine in T2DM. However, substantial heterogeneity in study designs, patient populations, study drugs, as well as in the assays and matrices employed, precluded the conduct of a formal meta-analysis.

### 4.1. Metformin

Metformin has been the undisputed first-line therapy for T2DM for decades, although it has recently been challenged by newer drug classes like SGLT-2 inhibitors and GLP-1 RAs. Nevertheless, it remains effective, inexpensive, with a good safety profile, and widely used. Its impact likely extends beyond glycemic control, offering benefits in cardiovascular prevention and—albeit to a lesser extent—potentially in oncology, contributing to longevity [67,68,69].

A potential mechanism underlying these effects is metformin’s impact on miRNA expression. Demirsoy et al. demonstrated that several miRNAs, namely let-7e-5p, let-7f-5p, miR21-5p, miR-24-3p, miR-26b-5p, miR-126-5p, miR-129-5p, miR-130b-3p, miR-146a-5p, miR-148a-3p, miR-152-3p, miR-194-5p, miR99a-5p, were downregulated after metformin administration in treatment-naïve T2DM patients. Clinical correlates were not analyzed [51].

However, at least some of these downregulated miRNAs have been suggested to play roles in carbohydrate metabolism and diabetic complications, based on findings from a limited number of animal and human studies. For instance, miRNA let-7e-5p modulates basal GLP-1 secretion [70], miR-26b-5p and miR-148a-3p may contribute to the development of chronic diabetic complications [71,72]. In addition, miR-146a-5p and miR-152-3p are involved in inflammation [73,74]. However, not all miRNAs downregulated by metformin have a clearly defined mechanistic role in diabetes control. Nevertheless, numerous studies link these miRNAs to other effects of metformin, including prevention of muscle atrophy, modulation of cell apoptosis, as well as with improvements in cardiovascular and cancer outcomes [75,76,77,78,79,80,81,82]. Intriguingly, neuropathy, a side effect typically attributed to metformin–induced vitamin B 12 deficiency, may at least in part be explained by the downregulation of miR-130b [83].

Although the clinical studies discussed in this review are encouraging and can be interpreted in the context of previous research, the overall certainty of evidence on the impact of metformin on miRNA expression is low, based on three observational studies that carry a moderate to serious risk of bias due to potential confounding, selective reporting, and missing data.

Nevertheless, further studies with a long follow-up period are needed to establish whether therapeutic modulation of these miRNA shifts translates into predictable clinical outcomes.

### 4.2. GLP-1 RAs

Evidence regarding another class of antidiabetic drugs, GLP-1Ras, is more heterogeneous. Nevertheless, these drugs have been in clinical use for a couple of decades and have demonstrated beneficial effects beyond glucoregulation. They have been shown to have cardioprotective effects and a positive influence on renal function in patients with T2DM, as well as in other patient groups. In addition, some of these drugs are also approved for obesity treatment [9,69,84].

Formichi et al. reported that baseline expression of miR-21-5p, miR-24-3p, miR-223-3p, and miR-375-5p predicted favorable glycemic outcomes of GLP-1 RA treatment, while miR-15a-5p was associated with weight reduction [58]. Other evidence has confirmed the involvement of miR-24-3p, miR-223-3p, and miR-375-5p in glucose regulation, insulin secretion and insulin resistance [85,86,87]. In contrast, miR-21-5p has been linked to the angiogenesis [88]. The association between miR-15a-5p and obesity has also been supported by previous evidence [89].

Al Zamily observed upregulation of miR-146a and miR-222 following liraglutide treatment, both of which were negatively correlated with HbA1c and fasting glucose, alongside downregulation of miR-21 [64]. All of these mRNAs have been previously described as important regulators of blood glucose, both in preclinical and clinical settings [90,91,92].

Similarly, Giglio et al. found liraglutide increased circulating miR-27b, miR-130a, and miR-210, independently of metabolic parameters, while Liu et al. showed reductions in miR-203a-3p and miR-429, potentially contributing to cardiovascular protection [55,63]. The role of miR-27b remains uncertain, as it has not been linked to glucose regulation and diabetic complications in other research [93]. In contrast, the role of miR-130a appears more promising. Evidence suggests beneficial effects of this miRNA on vascular health, aligning with the overall positive vascular effects of liraglutide [94]. In addition, miRNA 203a-3p and miRNA 429 are involved in inflammatory pathways, suggesting a potential role in glucose regulation [95,96]. In contrast, Gaborit et al. and Iacobellis et al. found no significant changes in circulating miRNA with liraglutide [56,66]. These discrepancies may reflect differences in treatment duration, endpoints, or sample types, highlighting the need for larger and more standardized trials.

Although the evidence on GLP-1 RAs is limited and fragmented, it provides fresh insight that expands previous knowledge of the mode of action and clinical impacts of these agents. A substantial limitation is that the impact of weekly GLP-1 RAs with proven cardiovascular benefits on miRNA expression has been investigated in only a single observational study, and neither semaglutide nor tirzepatide has yet been studied.

Conclusively, this review includes six studies on GLP-1 RAs (two of which are randomized controlled trials); however, the mixed directionality of miRNA changes, inconsistent endpoints, and indirect evidence for clinical translation support only a low overall certainty of evidence.

### 4.3. SGLT2 Inhibitors

SGLT-2 inhibitors are a relatively new class of antidiabetic drugs that exert effects beyond glucose regulation. Similar to GLP-1RAs, they have demonstrated cardioprotective properties and beneficial effects on renal function [9,69].

SGLT2 inhibitors also appear to consistently modulate miRNAs linked to inflammation and vascular function. Pehlivan et al. reported that dapagliflozin reduced miR-21, miR-141, and miR-377, with baseline miR-21 correlating with HbA1c reduction [65]. The clinical effect of these miRNA changes can contribute to improved beta cell function and inhibited hepatic gluconeogenesis, as previously described [92,97]. Although the role of miRNA-141 has been implicated in diabetes control, such a correlation was not confirmed in this study [98,99]. However, evidence from other research suggests that miR-377 exerts a positive effect on the heart and blood vessels [100,101,102,103].

Solini et al. observed upregulation of miR-30e-5p and downregulation of miR-199a-3p following dapagliflozin treatment, although without detectable vascular functional improvements [54]. Pathways mediated by miR-30e-5p are thought to be protective against diabetic nephropathy, a finding supported by animal studies, but not confirmed clinically in this research [102]. Furthermore, miRNA-199a-3p has been implicated in the pathogenesis of chronic diabetic complications [104]. Mone et al. demonstrated that empagliflozin reduced miR-21 and miR-92, both associated with endothelial dysfunction, suggesting potential vascular protective effects beyond glucose lowering [60,105,106].

Taken together, these studies indicate that SGLT2 inhibitors may provide epigenetic benefits relevant to both metabolic and cardiovascular health. The overall level of certainty remains low, although there is an RTC and two observational studies. However, the open-label design of the RCT and the serious risk of bias in the observational studies resulted in a low overall certainty of evidence.

### 4.4. Thiazolidinediones

Thiazolidinediones are a class of antidiabetic agents with evidence of improving vascular health. However, their clinical use may be limited by fluid retention, weight gain, heart failure, and peripheral bone fractures. They have also been shown to be effective in the treatment of NAFLD [107,108].

Pioglitazone has been shown to consistently influence circulating miRNA expression. Nunez Lopez et al. reported significant downregulation of multiple miRNAs, including miR-7-5p, miR-20a-5p, and miR-374b-5p, in parallel with improvements in HbA1c and insulin resistance [59]. The potential role of miR-7-1-5p as a marker for treatment response in type 2 diabetes remains under investigation, since some research claims that this mRNA is downregulated in patients with T2DM compared to healthy subjects [109]. In contrast, miR-20a-5p has been associated with insulin resistance, whereas circulating exosomal miR-20b-5p is elevated in type 2 diabetes and may impair insulin action in human skeletal muscle, suggesting that downregulation with pioglitazone could be clinically beneficial [110]. Interestingly, downregulation of miR-374b-5p has been linked to heart failure, a finding that was also noticed in this study [111]. Changes in miR-92a-3p appear non-specific, as it is also downregulated in patients treated with metformin [112].

Another miRNA implicated in vascular health is miR-195-5p, which facilitates endothelial dysfunction by inhibiting vascular endothelial growth factor A. Its downregulation by pioglitazone may therefore exert protective effects with clinical relevance [113].

Hong et al. reported increases in circulating miR-24, associated with reduced coronary neointimal hyperplasia and improved endothelial function [50]. These findings suggest that TZDs modulate both metabolic and vascular pathways through miRNA regulation, providing a mechanistic explanation for their vascular effects. Evidence shows that miR-24 appears protective against vascular complications [114].

Intriguingly, Redling et al. identified elevated miRNA-122-5p levels and low miRNA-431-5p and miRNA let-7g-5p levels as predictive markers for thiazolidindione treatment failure in young patients [62].

Collectively, these findings suggest that thiazolidinediones modulate both metabolic and vascular pathways through miRNA regulation, providing also a mechanistic explanation for their vascular effects. Although some miRNA effects appear context-dependent, the evidence highlights the potential of circulating miRNAs as both predictive biomarkers and mediators of TZD action. The overall certainty of evidence for this drug class remains low-to-moderate due to potential confounding, selective reporting, and missing data, while the RCT raised some concerns related to randomization and its open-label design.

### 4.5. DPP-4 Inhibitors

DPP-4 inhibitors are a class of antidiabetic agents with a favorable safety profile. In addition to improving glycemic control, they have been associated with reductions in albuminuria in patients with diabetes. To date, however, they are not considered to have cardioprotective effects [69,115].

Data on the effects of DPP-4 inhibitors on miRNA expression are mixed. Catanzaro et al. found that miR-126-3p and miR-223 were associated with favorable responses to sitagliptin, while miR-378 was linked to poor responses in elderly patients [52]. Interestingly, in other research high miR-126-3p levels were associated with cardiovascular events in a general population [116]. In diabetic nephropathy, miR-223 is downregulated, and miR-223-3p has been shown to mediate the diabetic kidney disease progression by targeting IL6ST/STAT3 pathway, indicating protective mechanisms against diabetic kidney disease [117,118]. Furthermore, overexpression of miR-378 is associated with low adiponectin levels, suggesting a potential mechanism for increased insulin resistance that may reduce the treatment response [119].

In contrast to Catanzaro, Cho et al. observed no significant urinary exosomal miRNA differences between patients on DPP-4 inhibitors and those on sulfonylureas, except for overexpression of miR-23a-3p in type 2 diabetic patients compared with healthy volunteers [57]. These discrepancies may reflect differences in biological sample types (serum vs. urine), populations studied, or treatment durations.

Similarly, Tain et al. reported that miR-210 overexpression negatively correlated with metabolic control [61]. Intriguingly, miR-210 may have deleterious effects on endothelial progenitor cells in newly diagnosed type 2 diabetes [120], compounding the harmful impact of hyperglycemia and endothelial function [121]. However, other studies found no difference in miR-210 expression between treatment groups [122].

In summary, while DPP-4 inhibitors show benefits on glycemic control and albuminuria, their effects on miRNA profiles are variable and widely not understood. Three studies have been identified (one of which is an RCT); however, the lack of blinding and selective reporting lowered the overall certainty of evidence.

### 4.6. Insulin Therapy

Insulin remains an important antidiabetic agent. Among patients with T2DM, it is indicated for those with reduced insulin secretion to improve glucose control and decrease the risk of acute hyperglycemic diabetic complications [123].

Insulin itself can reshape the miRNA landscape. Nunez Lopez et al. demonstrated that baseline miR-145-5p and miR-29c-3p predicted glycemic response to short-term intensive insulin therapy [53]. miR-29c-3p has been associated with hyperglycemia, renal fibrosis, and apoptosis. In vitro, the lncRNA TUG1/miR-29c-3p/SIRT1 axis regulates endoplasmic reticulum stress-mediated injury in renal epithelial cells in a diabetic nephropathy model [124]. Similarly, overexpression of miR-145 has been linked to diabetic complications [125]. Furthermore, responders to insulin therapy in the mentioned study exhibited longitudinal changes in miR-138-5p, miR-192-5p, and miR-320b, which correlated with improved β-cell function and insulin sensitivity [50]. This suggests that miRNAs not only predict insulin response but also serve as dynamic markers of treatment-driven pathophysiological adaptation. miR-138-5p has been reported to decrease insulin secretion, with obesity-induced adipocytes promoting diabetes through exosomal miR-138-5p-mediated regulation of β-cell function [126]. miR-192-5p is involved in diabetic retinopathy, as its upregulation inhibits the ELAVL1/PI3Kδ axis and attenuates microvascular endothelial cell proliferation, migration, and angiogenesis [127]. Other investigated miRNAs also highlight cardiovascular and microvascular risks. Downregulation of miR-195-5p inhibits endothelial-mesenchymal transition and myocardial fibrosis in diabetic cardiomyopathy by targeting Smad7 and the TGF-β1/Smads/Snail pathway [128]. Low serum miR-320b has been identified as a novel indicator of carotid atherosclerosis [129]. Additionally, let-7a-5p has been implicated in diabetic retinopathy through miRNA-mRNA regulatory networks [130].

In conclusion, insulin therapy improves glycemic control and modulates multiple miRNAs, which may serve as predictive biomarkers and mechanistic mediators of metabolic, microvascular, and cardiovascular adaptations in T2DM. However, with only a single observational study available, the overall certainty of evidence remains low due to a serious risk of bias, primarily arising from post hoc classification of responders and non-responders and potential confounding, despite the use of rigorous laboratory and statistical methods.

### 4.7. Other Therapies

Additional therapies have also been shown to influence circulating miRNAs. Tian et al. and Cho et al. used sulfonylureas as comparators in their studies [57,61]. Although some changes in miRNA expression were observed, these differences were not significant compared with the comparator groups. Overall, while additional antidiabetic therapies may modulate circulating miRNAs, current evidence suggests that these effects are modest and not consistently different from those observed with standard comparator treatments such as DPP-4 inhibitors. Therefore, the overall certainty of evidence remains low.

### 4.8. Overview of miRNAs Affected by Currently Approved Diabetes Drugs

A brief overview summarizing the specific miRNAs affected by diabetes pharmacotherapy and their biological effects is presented in Table 4.

## 5. Future Directions

The findings presented in this review highlight miRNAs as both promising biomarkers and potential therapeutic targets in T2DM management. Nonetheless, several critical steps are required to translate these early insights into clinical practice.

Robust, multi-center, randomized, and longitudinal studies with standardized methodologies are essential to confirm the predictive and prognostic value of specific miRNAs across diverse populations.

Furthermore, large interventional studies are needed to clarify the mechanistic role of individual miRNAs in mediating drug effects. While associations between miRNA expression and glycemic or cardiovascular outcomes have been described, causal links remain uncertain. Preclinical models and integrative approaches combining transcriptomics, proteomics, and metabolomics could help delineate whether miRNA modulation actively drives therapeutic benefits or merely reflects treatment response.

Finally, miRNAs may represent therapeutic targets. Antagomirs or miRNA mimics directed against disease-relevant pathways could provide novel adjunctive treatments for T2DM, particularly in individuals with suboptimal responses to existing therapies. Future clinical trials should explore whether modifying miRNA expression enhances treatment efficacy or reduces long-term complications.

## 6. Strengths and Limitations

The evidence summarized in this review offers notable strengths. The inclusion of interventional clinical studies provides valuable insights into causal relationships between glucose-lowering therapies and miRNA modulation, moving beyond purely observational associations. By analyzing a wide range of therapeutic classes—including metformin, GLP-1 receptor agonists, SGLT2 inhibitors, TZDs, DPP-4 inhibitors, and insulin—the reviewed literature highlights both shared and drug-specific miRNA signatures. This breadth allows comparison across different mechanisms of action and suggests that miRNA regulation may serve as a common pathway through which drug classes exert metabolic and other clinical benefits. Additionally, several studies explored longitudinal changes in miRNA expression, strengthening the case for their utility as dynamic biomarkers that reflect treatment response rather than static indicators of disease.

Despite these promising findings, important limitations temper the conclusions. Most studies were limited by small sample sizes, reducing statistical power and generalizability. Considerable heterogeneity in patient populations, disease duration, and comorbidities further complicates comparisons across studies. Methodological variability—including differences in biological sample types (plasma, serum, urine) may introduce significant bias and may explain inconsistent results. Moreover, the relatively short duration of most studies makes it difficult to determine whether observed miRNA changes translate into long-term clinical benefits. Another limitation is the lack of mechanistic validation; while many miRNAs have been associated with treatment response, their direct role in mediating drug effects remains unclear. In addition, possible language bias needs to be addressed, as four additional interventional studies in other languages were identified (three in Chinese and one in Ukrainian) [131,132,133,134]. Although the overall results were statistically significant, these studies were relatively small, recruiting a total of 137 participants with T2DM, of whom 66 received the study drug. The study drugs included metformin, dapagliflozin, and insulin, while various miRNAs were analyzed (miR-423-5p, hsa-miR-29a-3p, hsa-miR-133a-5p, hsa-miR-21-5p, hsa-miR-30c-5p, hsa-miR-1-3p, miR-let-7b-5p, miR-182-5p, miR-200c-3p, and miR-126) in different patient populations, including patients with T2DM with heart failure, and chronic kidney disease. Only a single trial was a randomized clinical trial. Compared with the English-language studies, these non-English studies had a lower proportion of randomized controlled trials and smaller sample sizes. The interventions and outcomes assessed in the English studies were largely similar, and no contradictory findings were observed.

## 7. Conclusions

Current evidence for personalized pharmacotherapy in type 2 diabetes remains limited and heterogeneous. Although patterns are emerging—such as the vascular-protective signature of SGLT2 inhibitors, the metabolic and cardiovascular effects of TZDs, and predictive roles for GLP-1RA and insulin responses—most available studies are small and exploratory, and vary considerably in study design, outcomes, and patient populations.

Progress toward clinical translation will require standardized profiling methods, longer follow-up periods, and larger patient cohorts.

Taken together, while current evidence demonstrates a low to low-to-moderate level of certainty, it nonetheless provides a rationale for further research. Robust, large-scale, and well-controlled clinical studies with standardized methodology are required before miRNAs can be integrated into routine precision medicine for T2DM.

In summary, miRNAs represent an emerging field with potential to reshape precision medicine in diabetes care. Realizing this promise will depend on coordinated translational efforts that integrate mechanistic studies, biomarker validation, and well-designed interventional trials.

## Figures and Tables

**Table 1:** Summary of interventional studies on miRNA in T2DM patients.

Reference	Study Population	Sample Type	miRNA Platform/ Method	Normalization/ Hemolysis Control	Intervention/Control Group	Outcome	Guidelines Adherence
Hong et al., 2015 [50]	72 patients with T2DM and CAD Age: 45–75 years HbA1c: no data	serum	RT-qPCR/TaqMan microRNA assay	data not provided/data not provided	randomly assigned to pioglitazone or placebo in a 1:1 ratio follow-up: 9 months	neointimal volume (mm^3^, measured by optical coherence tomography) was significantly lower in the pioglitazone group increased FMD (mm, color Doppler) in the pioglitazone group significant increases in circulating miRNA-24 in the pioglitazone group	70%
Demirsoy et al., 2018 [51]	47 patients with drug-naive T2DM Age: males 54 ± 11 years, females: 49 ± 16 years HbA1c: males 7.2 ± 3.6%, females: 7.6 ± 4.2%	plasma	qRT-PCR/Fluidigm BioMark microfluidic array	global mean normalization/data not provided	metformin follow-up: 3 months no control group	let-7e-5p, let-7f-5p, miR21-5p, miR-24-3p, miR-26b-5p, miR-126-5p, miR-129-5p, miR-130b-3p, miR-146a-5p, miR-148a-3p, miR-152-3p, miR-194-5p, miR99a-5p were significantly downregulated	75%
Catanzaro et al., 2018 [52]	40 T2DM patients treated with metformin age > 65 years HbA1c: 7.5–9.0%	plasma	qRT-PCR/TaqMan Low-Density Array v3.0	Global mean normalization/visual inspection + expression check of hemolysis-sensitive miRNAs	sitagliptin 100 mg follow up 15 months no placebo group	miR-378 associated with a treatment failure miR-126-3p and miR-223, associated with favorable glycemic responses to sitagliptin (HbA1c < 7.5% or HbA1c reduction >0.5%)	70%
Nunez Lopez et al., 2019 [53]	24 insulin-naive patients with T2DM Age: responders: 62.8 ± 8.3 years non-responders: 56.0 ± 10.2 years HbA1c: responders: 6.7 ± 0.4% non-responders: 7.6 ± 1.2%	plasma	qRT-PCR/TaqMan Array MicroRNA Cards	Spike-in + quantile normalization/ratio-based molecular control	basal insulin detemir and premeal insulin aspart follow-up: 4 weeks no control group	baseline levels of circulating miR-145-5p, miR-29c-3p, and HbA1c predicted favorable response to insulin treatment (fasting blood glucose < 7 mmol/L) significant longitudinal changes due to insulin treatment in the circulating levels of miR-138-5p, miR-192-5p, miR-195-5p, miR-320b, and let-7a-5p characterized the responder group and correlated with the changes in measures of beta-cell function (ISSI-2, %) and insulin sensitivity (HOMA-IR, %)	70%
Solini et al., 2019 [54]	40 patients with T2DM and hypertension age: 40–75 years HbA1c: <8.0%	serum	qRTPCR/TaqMan Advanced MicroRNA Assays	multiple endogenous reference miRNAs/data not provided	randomly assigned to dapagliflozin or hydrochlorothiazide in a 1:1 ratio follow-up: 4 weeks no placebo group	dapagliflozin, but not HCT, significantly up-regulated miR30e-5p and down-regulated miR199a-3p no changes in FMD or carotid-femoral pulse-wave velocity (mm, color Doppler)	60%
Giglio et al., 2020 [55]	25 patients with T2DM on metformin age: 64.6 ± 8.4 years HbA1c: 8.3% (IQR: 0.6%)	serum	qRT-PCR/SYBR Green–based detection	Single endogenous control/data not provided	liraglutide follow-up: 4 months no control group	levels of miRNA-27b, miRNA-130a and miRNA-210a were significantly increased in the liraglutide group, independently of metabolic parameters (blood glucose-mmol/L, HbA1cin %, blood lipids in mmol/L)	50%
Gaborit et al., 2020 [56]	49 obese T2DM patients treated with metformin and/or insulin secretagogues Age: study group 52 ± 10 years control group: 55 ± 8 years HbA1c: Study group: 7.43% Control group: 7.57 ± 1.56%	Serum	qRT-PCR/ miScript miRNA PCR Array	cel-miR-39 exogenous control/data not provided	liraglutide vs. placebo follow-up: 4 weeks	no effect on circulating miRNAs	35%
Cho et al., 2021 [57]	57 patients with T2DM age: healthy volunteer: 42.3 ± 11.5 DPP4 inhibitor: 53.2 ± 11.6 sulfonylurea: 53.7 ± 11.9 HbA1c: DPP4 inhibitor: 6.76 ± 0.94% sulfonylurea: 7.21 ± 1.34%	Urine	qRT-PCR/NanoString nCounter Human v3 miRNA Expression Assay and TaqMan Advanced miRNA qPCR	NanoString internal normalization/not applicable	DPP-4 inhibitor group (*n* = 34) sulfonylurea group (*n* = 23) healthy volunteers (*n* = 7) no placebo group follow-up: 3 months	no significant difference in miRNA expression between the DPP-4 inhibitor and sulfonylurea groups. miR-23a-3p was significantly overexpressed in the diabetes group	60%
Formichi et al., 2021 [58]	26 T2DM patients treated with metformin age: 60.3 ± 10.3 (35–79) years HbA1c: 7.7 ± 0.58 (6–8.8)%	Plasma	qRT-PCR/TaqMan miRNA qRT-PCR	miR-191-5p used as endogenous control/data not provided	GLP-1 RAs: liraglutide (*n* = 8) or dulaglutide (*n* = 18) no control group follow-up: 12 months	miR-21-5p, miR-24-3p, miR-223-3p and miR-375-5p at baseline associated with favorable glycaemic outcome (HbA1c < 7%) higher baseline miR-15a-5p expression was associated with weight loss > 5%	70%
Nunez Lopez et al., 2022 [59]	24 subjects with well controlled T2DM treated with diet and/or metformin age: 62.50 [IQR: 48.50, 65.25] years in the study group 61.50 [IQR 56.00, 66.00] years in the placebo group HbA1c < 7.0%	Circulating extracellular vesicles, Adipose tissue	TaqMan MicroRNA Array (ThermoFisher ViiA-7) and RNeasy Lipid Tissue Mini Kit	miR-126 & miR-30b/data not provided	participants randomized to either placebo or pioglitazone in a 1:1 ratio follow-up: 12 weeks	association of circulating miR-374b-5p changes with changes in HbA1c (%), fasting plasma glucose (mmol/L), and insulin resistance parameters (Si, AIRg, Sg) circulating miR-7-5p, miR-20a-5p, miR-92a-3p, miR-195-5p, and miR-374b-5p significantly downregulated in response to pioglitazone miR-195-5p upregulated in response to pioglitazone in fat tissue	70%
Mone et al., 2023 [60]	30 frail older adults with T2DM and HFpEF Age > 65 years HbA1c: no data	Plasma	RT-qPCR/RT: miRCURY LNA Universal RT microRNA PCR kit	miR-320a and miR-423-5p/data not provided	participants assigned to empagliflozin, metformin or insulin in a 1:1:1 ratio no placebo group 10 healthy controls follow-up: 3 months	miR-21 and miR-92 were significantly reduced in the empagliflozin group compared to other groups	55%
Tian et al., 2023 [61]	86 T2DM with NAFLD treated with diet and/or metformin age 40–70 years; body mass index (BMI) of 18.0–25.0 kg/m^2^ HbA1c: no data	Serum	qRT-PCR, SYBR Green	U6 snRNA as endogenous control/data not provided	linagliptin (5 mg/daily) vs. glimepiride (2 mg/daily) no placebo group follow-up: 6 months	miR-210 positively correlated with fasting blood glucose level and 2 h post-breakfast blood glucose level (mmol/L) miR-220 negatively correlated with fasting insulin level miR-210 ALT/AST ratio positively correlated with miR-210 expression no difference between study groups in miR-210 expression	50%
Redling et al., 2024 [62]	365 youth with T2DM Age: 10–17 years T2DM duration < 2 years BMI ≥ 85th percentile for age HbA1c: no data	Plasma	NanoString nCounter Human v3 miRNA Panel and TaqMan Advanced miRNA Assays	data not provided/data not provided	participants randomily assigned to: metformin, metformin + rosiglitazone, or metformin + lifestyle intervention no placebo group follow-up 2–10 years	high miRNA-122-5p and low miRNA-431-5p and miRNA-let-7g-5p predicted treatment failure specified as the outcome measure (HbA1c ≥ 8% for at least 6 months or inability to wean from insulin after a metabolic decompensation)	60%
Liu et al., 2024 [63]	63 patients with T2DM and CAD matched with 63 patients with T2DM without CAD Age: no data HbA1c: 8.64 ± 2.15%	Serum	qRT-PCR using Qiagen miScript system and SYBR Green	Exogenous spike-in control: cel-miR-39/data not provided	liraglutide no placebo group follow-up: 12 months	miR-203a-3p and miR-429 decreased from baseline levels improvements in clinical metabolic parameters (HbA1c-%, blood lipids–mg/dL)	75%
Al Zamily, 2025 [64]	60 patients recently diagnosed with T2DM Age: 46.04 ± 13.71 years HbA1c: 7.89 ± 0.57%	Blood	qRT-PCR/Two-step qRT-PCR on Rotor-Gene Q thermocycler (Qiagen/Thermo Fisher)	data not provided/data not provided	liraglutide a 1.5 mg daily no control group follow-up: 6 months	miRNA-146a and miRNA-222 levels were upregulated miRNA-21 was downregulated miRNA-146a and miRNA-222 negatively correlated with HbA1c (%) and fasting blood glucose, (mmol/L) while miRNA-21 correlated positively	50%
Pehlivan et al., 2025 [65]	47 T2DM patients with diabetic nephropathy age: 54.09 ± 8.48 years HbA1c: 9.85 ± 1.83%	Plasma	qRT-PCR/D3EAL miRNA qPCR System kit	data not provided/data not provided	dapagliflozin no control group follow-up: 60 days	reductions in miRNA-21, miRNA-141, and miRNA-377 levels, baseline miRNA-21 levels correlated with HbA1c (%) reductions	90%
Iacobellis et al., 2025 [66]	38 patients with T2DM and CAD age: study group: 63.7 ± 7.7 years control group: 65 ± 11.6 years HbA1c: 6.9 ± 1.1% vs. 7.2 ± 1.3%	Epicardial and Subcutaneous Adipose Tissue	qRT-PCR, TaqMan primers	U6 (for miRNAs), GAPDH (for mRNA)/data not provided	liraglutide vs. placebo follow-up: 12 weeks	miR16, miR155 and miR181a were significantly higher in epicardial adipose tissue than in subcutaneous adipose tissue no significant changes in the liraglutide group	75%

**Table 2:** Risk of Bias evaluation of interventional studies on miRNA in T2DM patients.

	Design	Tool Used	Domain: 1	Domain: 2	Domain: 3	Domain: 4	Domain: 5	Domain: 6	Domain: 7	Overall Risk	
Hong et al., 2015 [50]	RCT	RoB2	some concerns	some concerns	some concerns	low	low	n.a.	n.a.	some concerns	Mainly due to randomization and open-label design
Demirsoy et al., 2018 [51]	pre-post intervention study without randomization or a control group	ROBINS-I	serious	moderate	low	moderate	serious	low	serious	serious	Confounding (no control group) Potential selective reporting of significant miRNAs
Catanzaro et al., 2018 [52]	non-randomized, uncontrolled intervention study	ROBINS-I	serious	moderate	low	moderate	some concerns	low	serious	serious	Confounding (no control group, baseline differences) Selective outcome reporting (multiple miRNAs tested; only significant ones reported)
Nunez Lopez et al., 2019 [53]	non-randomized, single-arm intervention study	ROBINS-I	serious	moderate	low	low	low	low	moderate	serious	Serious risk of bias, mainly due to post hoc classification of responders/nonresponders and potential confounding, despite rigorous lab and statistical methods.
Solini et al., 2019 [54]	RCT	RoB2	low	some concerns	low	low	low	n.a.	n.a.	low	open-label design
Giglio et al., 2020 [55]	prospective, single-arm, interventional study	ROBINS-I	serious	moderate	low	low	low	moderate	serious	serious	lack of control group, confounding, and selective outcome reporting
Gaborit et al., 2020 [56]	RCT	RoB2	low to moderate	moderate	low	low	low to moderate	n.a.	n.a.	moderate	short duration, open-label design, and multiple exploratory outcomes
Cho et al., 2021 [57]	prospective observational study	ROBINS-I	moderate to serious	moderate	low	low	low to moderate	low	moderate	moderate	potential confounding and the small sample size for primary miRNA profiling.
Formichi et al., 2021 [58]	prospective, single-arm, observational pilot study	ROBINS-I	moderate	moderate	low	low	low to moderate	low	moderate	moderate	small sample size, lack of control group, and potential confounding.
Nunez Lopez et al., 2022 [59]	RCT	RoB2	low	low	low	low	low to moderate	n.a.	n.a.	low	small sample size
Mone et al., 2023 [60]	prospective, non-randomized, observational interventional study	ROBINS-I	serious	moderate	low	low to moderate	low	low	moderate	serious	confounding due to non-randomized allocation
Tian et al., 2023 [61]	RTC	RoB2	low	low to moderate	low	low	low to moderate	n.a.	n.a.	low to moderate	lack of blinding and selective reporting.
Redling et al., 2024 [62]	randomized clinical trial (RCT) in youth-onset T2D, but the microRNA analyses were largely observational within the trial	ROBINS-I	moderate	low	low	low	low to moderate	low	moderate	moderate	confounding, selective reporting, and missing data introduce moderate concerns.
Liu et al., 2024 [63]	observational study with pre-post intervention measurements	ROBINS-I	serious	moderate	low	low	low to moderate	low	moderate	serious	confounding due to disease group differences (T2DM with vs. without CAD).
Al Zamily, 2025 [64]	single-arm prospective interventional study	ROBINS-I	serious	low to moderate	low	low to moderate	low	low	moderate	serious	lack of a control group, leading to high risk of confounding
Pehlivan et al., 2025 [65]	retrospective, single-arm pre/post study	ROBINS-I	serious	moderate	low	low	low	low	moderate	serious	lack of control group and confounding, plus potential selective outcome reporting
Iacobellis et al., 2025 [66]	RTC	RoB2	low	low	low	low	low	n.a.	n.a.	low	none

**Table 3:** GRADE assessment of evidence for the effects of antidiabetic drugs on miRNA expression.

Drug Class	Evidence Base	GRADE Level	Rationale
metformin	mostly small RCTs/observational studies	low	consistent downregulation of multiple miRNAs, mechanistic links to β-cell function and inflammation, but limited sample size and follow-up
GLP-1 RAs	small heterogeneous trials	low	mixed directionality of miRNA changes, inconsistent endpoints, indirect evidence for clinical translation
SGLT2 inhibitors	small RCTs/observational studies	low	some consistent miRNA changes related to inflammation/vascular protection, limited clinical correlation
TZDs	small RCTs/observational studies	low to moderate	miRNA changes linked to HbA1c and endothelial function, but sample sizes limited, some context-dependent results
DPP-4 inhibitors	small RTCs/observational studies	low	highly variable miRNA effects, inconsistent correlations with clinical outcomes
insulin therapy	small RCT	low	predictive miRNAs identified, dynamic changes observed, but mostly short-term studies

**Table 4:** Predominant miRNAs, canonical functions and clinical links for analysed drug classes.

Drug Class	Predominant miRNAs	Canonical Functions/Mechanistic Roles	Translational/Clinical Link
Metformin	let-7e-5p, let-7f-5p, miR-21-5p, miR-24-3p, miR-26b-5p, miR-126-5p, miR-129-5p, miR-130b-3p, miR-146a-5p, miR-148a-3p, miR-152-3p, miR-194-5p, miR-99a-5p	Carbohydrate metabolism (let-7e-5p), chronic diabetic complications (miR-26b-5p, miR-148a-3p), inflammation (miR-146a-5p, miR-152-3p), apoptosis, muscle atrophy, vascular and cancer effects	Improved HbA1c, potential cardiovascular and oncologic benefits; neuropathy risk via miR-130b downregulation
GLP-1 RAs	miR-21-5p, miR-24-3p, miR-223-3p, miR-375-5p, miR-15a-5p, miR-146a, miR-222, miR-27b, miR-130a, miR-210, miR-203a-3p, miR-429	Glucose regulation, insulin secretion/resistance (miR-24-3p, miR-223-3p, miR-375-5p), angiogenesis (miR-21-5p), obesity modulation (miR-15a-5p), inflammation (miR-203a-3p, miR-429), vascular health (miR-130a)	Glycemic control, weight reduction, cardiovascular and renal protective effects; mechanistic insights into miRNA modulation of glucose and vascular pathways
SGLT2 inhibitors	miR-21, miR-141, miR-377, miR-30e-5p, miR-199a-3p, miR-92	Inflammation (miR-21, miR-377), vascular protection (miR-377, miR-30e-5p), chronic complications (miR-199a-3p), endothelial function (miR-92)	Improved HbA1c, potential β-cell and cardiovascular protection, nephropathy prevention, limited evidence on vascular functional outcomes
Thiazolidinediones (TZDs)	miR-7-5p, miR-20a-5p, miR-374b-5p, miR-92a-3p, miR-195-5p, miR-24, miR-122-5p, miR-431-5p, let-7g-5p	Insulin resistance (miR-20a-5p), endothelial dysfunction (miR-195-5p), vascular protection (miR-24), heart failure (miR-374b-5p), predictive markers of response (miR-122-5p, miR-431-5p, let-7g-5p)	Improved HbA1c, insulin sensitivity, endothelial function; predictive biomarkers for TZD efficacy and vascular protection
DPP-4 inhibitors	miR-126-3p, miR-223-3p, miR-378, miR-23a-3p, miR-210	Glycemic response (miR-126-3p, miR-223-3p), diabetic kidney disease progression (miR-223-3p), insulin resistance (miR-378), endothelial progenitor cell function (miR-210)	HbA1c improvement, albuminuria reduction; miRNA effects variable and less consistent; potential vascular and renal implications
Insulin therapy	miR-145-5p, miR-29c-3p, miR-138-5p, miR-192-5p, miR-320b, let-7a-5p, miR-195-5p	β-cell function and insulin sensitivity (miR-138-5p, miR-192-5p), hyperglycemia, renal fibrosis, apoptosis (miR-29c-3p), cardiovascular and microvascular complications (miR-195-5p, let-7a-5p), diabetic complications (miR-145-5p)	Predictive markers of insulin response; dynamic indicators of treatment-driven metabolic, microvascular, and cardiovascular adaptation
Other therapies (e.g., sulfonylureas)	Variable; modest changes observed	Limited mechanistic data	No consistent miRNA effects relative to standard comparator treatments

## Data Availability

The raw data supporting the conclusions of this article will be made available by the authors on request.
